# Shoulder muscle EMG activity during push up variations on and off a Swiss ball

**DOI:** 10.1186/1476-5918-5-7

**Published:** 2006-06-09

**Authors:** Gregory J Lehman, Brandon MacMillan, Ian MacIntyre, Michael Chivers, Mark Fluter

**Affiliations:** 1 Department of Graduate Studies, Canadian Memorial Chiropractic College, Toronto, ON, Canada; 2 Undergraduate Department, Canadian Memorial Chiropractic College, Toronto, ON, Canada

## Abstract

**Background:**

Surface instability is a common addition to traditional rehabilitation and strength exercises with the aim of increasing muscle activity, increasing exercise difficulty and improving joint proprioception. The aim of the current study was to determine if performing upper body closed kinetic chain exercises on a labile surface (Swiss ball) influences myoelectric amplitude when compared with a stable surface.

**Methods:**

Thirteen males were recruited from a convenience sample of college students. Surface electromyograms were recorded from the triceps, pectoralis major, latissimus dorsi, rectus abdominis and external oblique while performing push up exercises with the feet or hands placed on a bench and separately on a Swiss ball. A push up plus exercise was also evaluated with hands on the support surface.

**Results and discussion:**

Not all muscles responded with an increase in muscle activity. The pectoralis major muscle was not influenced by surface stability. The triceps and rectus abdominis muscles showed increases in muscle activity only when the hands were on the unstable surface. The external oblique muscle was only influenced by surface stability during the performance of the push up plus exercise. No muscle showed a change in activation level when the legs were supported by the Swiss ball instead of the bench.

**Conclusion:**

Muscle activity can be influenced by the addition of surface instability however an increase in muscle activity does not influence all muscles in all conditions. The relationship between the participant's center of mass, the location of the unstable surface and the body part contacting the Swiss ball may be important factors in determining the muscle activation changes following changes in surface stability.

## Introduction

Exercise balls, wobble boards and other labile surfaces commonly replace stable surfaces during the performance of resistance training exercises for both injury management and performance improvement. A common assumption is that an unstable surface places an increased demand on the neuromuscular system to stabilize articular joints which may have been created more unstable due to the labile surface. The purported benefits of training with this instability are improvements in joint proprioception and greater muscle activation requirements. Research investigating ankle disc training has shown improvements in proprioception and muscle reflex latency time [1,2] following the training regime and reduction in injury prevalence [3]. Similar improvements in joint proprioception have been documented in unstable shoulders following rehabilitation therapy using unstable surfaces [4].

It is often assumed that performing a resistance exercise on unstable surfaces results in greater muscle activity in an attempt to achieve joint stability. This assumption has mixed and somewhat sparse support. The majority of research has investigated the influence of labile surfaces on trunk muscle activity during trunk training exercises. Some research shows a consistent increase in selected (not all) trunk muscles during curl ups on an exercise ball [5], upper body exercises while seated on an exercise ball [6] and during unstable weighted squat movements while standing on semi inflated wobble discs [7]. Others have shown inconsistent changes across subjects with no statistical increase in muscle activity when replacing a Swiss ball for an exercise bench during resistance exercises for the upper body [6,8] and changes dependent upon centre of gravity location relative to the unstable surface during bridging/core stability exercises [9,10].

Anderson & Behm [11] documented the muscle activity of the primary movers and the force output during a chest press while lying on a Swiss ball (labile surface) and on a bench. The authors found a decrease in absolute force production on the labile surface when compared with the force produced on the stable surface; however, there was no difference in the amount of muscle activity between the two conditions. Suggesting that more muscle activation was required to achieve the same amount of force production on an unstable surface compared with a stable surface. This suggests that for the same amount of force production on an unstable surface compared with a stable surface requires a greater amount of muscle activation. An exercise similar to the chest press is the push up exercise. A review of the literature failed to provide any work documenting the influence of an unstable surface on the myoelectric activity of the shoulder and trunk muscles during push up exercises. This exercise assumes that the required force production during the push up will be consistent (due to gravity and body weight) regardless of the stability of the support surface. Therefore investigating this exercise allows us to determine if an unstable support surface necessitates greater muscle activation when the same force requirements are demanded across the same exercise performed on an unstable (Swiss ball) and stable surface (bench). It is the purpose of the current study to determine the effect of an unstable surface under the hands or under the feet during the push up and push up plus exercise on shoulder and trunk muscle activation levels.

## Methods

### Patient characteristics

Thirteen healthy males (average age in years (standard deviation) 26.3 (1.5), average height (standard deviation) 176.7 cm (4.99) and average weight (standard deviation) 79.6 kg (7.34) with greater than 6 months of weight training experience, without back pain or upper limb injuries were recruited from a convenience sample of college students. Participants were required to sign an information and informed consent form prior to the study approved by the institution's Research Ethics Board.

### Study protocol

To optimize EMG signal collection participants from a college population were recruited because of their athletic abilities and low subcutaneous fat. The myoelectric activity of the pectoralis major, latissimus dorsi, triceps, rectus abdomins and external oblique muscles were recorded during a series of different variations of the classic push up exercise.

### Data collection hardware characteristics

Disposable bipolar Ag-AgCI disc surface electrodes with a diameter of one cm were adhered over the muscle groups parallel to their fiber orientation in the muscle belly. Before the application of the electrodes the skin was shaved with a disposable razor and abraded with a cotton swab and alcohol. The electrodes were attached to 5 leads which were connected to an EMG data collection system (Bortec, Calgary AB). The myoelectric activity of the muscles was collected with customized software (Delys EMGWorks, Boston, MA, USA). Raw EMG was amplified between 1000 and 20,000 times depending on the subject. The amplifier had a CMRR of 10,000:1 (Bortec EMG, Calgary AB, Canada). Raw EMG was band pass filtered (10 and 1000 Hz) and A/D converted at 2048 Hz using a National Instruments data acquisition system.

### Maximum Voluntary Contractions (MVC)

In order to compare muscle activity across subjects and give biologically meaningful data maximal normalization contractions were performed for each muscle. This required the subject to maximally contract each muscle against the manual resistance provided by the experimenter. A maximal isometric contraction occurred twice for each muscle to ensure that an acceptable signal was recorded for each subject. The maximum muscle activity was calculated and recorded from a suitable maximum contraction and all subsequent muscle activity was expressed as a percentage of this maximum voluntary contraction (MVC).

### Electrode placement and MVC testing procedure

The triceps electrode was placed on the long head (middle of the muscle belly) between origin and insertion. The MVC saw the shoulder and elbow flexed to 90 degrees while the EMG was recorded during resisted elbow and shoulder extension. The pectoralis major electrode was placed four fingerbreadths below the clavical, medial to the anterior axillary border. With the elbow flexed 90 degrees and the shoulder abducted 75 degrees the subject performed a maximal palm press while the muscle activity was recorded. The latissimus dorsi electrodes were 3 finger fingerbreadths distal to and along the posterior axillary fold, parallel to the lateral border of the scapula. With the elbow extended and the arm abducted 30 degrees in the coronal plane and internally rotated, attempted maximum shoulder extension, adduction and internal rotation was resisted with the muscle activity recorded. The rectus abdominis electrode was placed 3 cm lateral to the umbilicus in a vertical orientation. With the participant supine, trunk flexion was resisted and the MVC was recorded. The external oblique electrode was placed 15 cm lateral to the umbilicus on a 45 degree inferior angle. With the subject lying supine with knees flexed 90 degrees, resisted side bending was recorded.

### Exercise protocol

Following the maximal voluntary contractions the participants were required to perform the following exercises in random order (arbitrarily determined by the experimenters). Participants performed the exercises identically across exercises. Three repetitions occurred for each exercise at the same rate. Participants began in the upright position when the EMG collection began. This position was held for 4 seconds. The eccentric (lowering) portion lasted 2 seconds. The patient then held the lowered position for 4 seconds. The concentric (raising) portion of the movement lasted for 2 seconds with a 4 second holding position once again in the upright position. Three repetitions were recorded during a 40 second collection period. An electrical trigger (foot switch) was used to mark the beginning of the first descent and the finish of the last repetition.

### Movement tasks

All movements were completed in a random order in a standardized position with the hands shoulder width apart with the subject's middle finger under the acromioclavicular joint. The bench height and exercise ball height were standardized and identical to each other. A minimum of 3 minutes of rest occurred between exercises to prevent the influence of fatigue on myoelectric amplitude changes. This rest period is similar and exceeds other studies investigating similar phenoma [5,7,9].

1. Push up with feet on an exercise bench.

2. Push up with feet on exercise ball.

3. Push up with hands on exercise bench.

4. Push up with hands on exercise ball.

5. Push up plus with hands on exercise bench: Starting in the push up position the participant rolls the shoulders forward (scapular protraction) and then lowers their body while allowing the shoulder blades to approximate (scapular retraction).

6. Push up plus with hands on exercise ball. Same movement as #5.

### EMG analysis

Both MVC data and myoeletric data from the exercises tasks were processed in the identical manner. Using EMG analysis software (EMGWorks, Delsys, Boston, MA) the myoelectrical was first demeaned (bias removed therefore the signal alternates around 0), then a root mean square technique (window of 150 ms and an overlap of 75 ms) was used to smooth the data thus providing a linear envelop of EMG activity. Using the electrical markings left by the foot switch trigger at the start and end of the movement the mean activity for the 3 repetitions was calculated. This mean activity was then expressed as a percentage of the peak activity found during the maximum voluntary contraction (MVC) for the corresponding muscle

### Statistical analysis

A series of paired *t-tests *were used to assess for the influence of the Swiss ball on muscle amplitude. Differences across the different exercises was not examined-only between the stable and unstable conditions.

## Results

Table 1 shows the average muscle activity and standard deviations for the 6 different exercises studied. The latissimus dorsi values were removed from the study as the first six participants analyzed showed primarily noise and little muscle activity during the performance of the push up. The triceps muscle was significantly influenced by the addition of the Swiss ball during the performance of the push up with the hands on the ball surface and during the push up plus with the hands on the ball surface. Both exercises showed a statistically significant increase in muscle activity when performed on the Swiss ball. Conversely, the triceps activity was not influenced when the Swiss ball replaced the bench under the participant's feet. The addition of the Swiss ball did not influence the muscle activity of the pectoralis major during all exercises. Similar to the triceps the Swiss ball addition resulted in a significant increase in the myoelectric activity of the rectus abdominis during push ups with the hands on the Swiss ball and the push up plus. There was no change in muscle activity when the feet were on the unstable surface. The external oblique was not influenced by the addition of the Swiss ball for either standard push up exercise. The external oblique showed a statistically significant increase when the Swiss ball was added during the push up plus exercise.

**Table 1:** Average trunk and shoulder muscle activity during the push up and push up plus exercises when performed on a Swiss ball and an exercise bench.

	**Triceps**		**Pectoralis Major**		**Rectus Abdominis**		**External Oblique**	
	**Bench**	**Ball**	**Bench**	**Ball**	**Bench**	**Ball**	**Bench**	**Ball**
**PUHands**	22.2 ± 8.8	*43.1 ± 17.3 p = .002	21.4 ± 11.8	26.65 ± 14.5 p = .341	13.4 ± 5.4	22.6 ± 8.6 p = .001	20.9 ± 15.6	24.07 ± 11.9 p = .1191
**PUFeet**	32.7 ± 17.5	29.9 ± 10.1 p = .355	30.4 ± 9.7	29.9 ± 7.6 p = .717	19.2 ± 6.9	19.6 ± 7.14 p = .742	27.79 ± 13.2	26.7 ± 9.8 p = .575
**PUPHands**	18.7 ± 10.3	*29.6 ± 14.7 p = .024	14 ± 7.2	21.4 ± 18.5 p = .079	14.9 ± 5.5	*25.4 ± 10.2 p = .011	19.2 ± 11.9	*23.9 ± 11.9 p = .0069

Muscle activity is expressed as a percentage of a maximum voluntary contraction (%MVC). A * indicates a statistically significant difference (p < .05) between the exercises using a Swiss ball or bench as the support surface (PUHands = Push up with hands on bench/ball, PUFeet = push up with feet on ball/bench and PUPHands = push up plus exercise with hands on ball/bench).

## Discussion

Replacing an exercise bench for a Swiss ball can increase muscle activity however, the effect is both task and muscle dependent. The triceps and rectus abdominis were the two muscles most affected by the addition of an unstable surface. The pectoralis major muscle – the primary mover – was not influenced by the addition of the Swiss ball during any push up variation.

It is not possible to conclude that surface instability automatically results in increases in muscle activity. Merely adding an unstable surface is insufficient to influence all muscles. Of note is that placing the feet on the unstable surface resulted in no changes in muscle activity in all of the muscles studied. Our findings suggest that the unstable surface needs to be under the hands in order to result in an assumed destabilization effect and a subsequent increase in muscle activity during push up variations. It also appears that the further the distance the centre of mass is above the base of support (when unstable) can also influence the trunk muscle activity. While we did not measure the position of the centre of mass in this study we believe it is safe to assume that the centre of mass of the subject is higher relative to the Swiss ball when the hands are on the Swiss ball than when the feet are on the Swiss ball considering that the feet are the highest body part during the push up when the feet are on the Swiss ball. A different result may occur if the feet are placed on the Swiss ball and the hands are on a bench of equal height to the Swiss ball. This vertical distance from the Swiss ball may be an important factor in determining which exercises will see changes in myoelectric activity with the addition of a Swiss ball. Of interest was the finding that the external oblique was not influenced by the Swiss ball during the standard push up; however, when the participants performed the push up plus which finds the arms extended and the participant's chest farther away from the Swiss ball the external oblique showed greater activity during the unstable condition. We assume in our study that the centre of mass is higher during the push up plus with the arms extended than during the push up exercise that saw the elbows flex and extend with no scapular protraction at the top of the push up. The finding that the distance between chest and hands on a Swiss ball influences trunk muscle activity has previously been reported by Marshall and Murphy [9] during bridging/front support exercises.

The lack of change in the pectoralis major muscle during the push ups on the different stability surfaces is interesting considering the dramatic change in the triceps muscle. This may be due to the differences between the joints and associated movements that the two muscles cross. There is greater redundancy in the motor control of muscles crossing the anterior shoulder. The joint is stabilized by a multitude of muscles (biceps brachii, anterior deltoid, rotator cuff) and shoulder adduction torque is also created by a number of muscles in addition to the pectoralis major. There is also a smaller range of motion compared with the elbow joint. The pectoralis major is a single joint muscle whereas the triceps brachii is a two joint muscle which has stability and movement demands both at the elbow and the shoulder possibly resulting in such a dramatic change in muscle activity when the Swiss ball replaced the bench during the push up. The pectoralis major may only be concerned with its primary movement and have a smaller role in responding to changes in stability which may be controlled by other muscles which influence the shoulder joint. In contrast, the triceps brachii is the main extensor of the elbow with little help from the anconeus. Due to its mechanical advantage relative to the length of the forearm it may also have difficulty in responding to changes in stability compared with pectoralis major. It should also be noted that there is often a range of responses as seen in previous research [8]. Not every individual responded in the same manner to a change in surface stability. It is possible that there are individual factors that modulate the response to surface stability which also suggests that training may be influence the response to instability. We don't know if the increase in muscle activity or lack of change is involuntary or can be subject to change with training and feedback.

A limitation to explaining our results is the lack of kinematic and kinetic information describing the push up variations in our study. Having information on the centre of mass and its relationship to the bases of support (feet and hands) in the different exercises may help explain the different results found when the feet were placed on the Swiss ball compared with the hands on the Swiss ball. An attempt was made in this study to control for joint posture and the influence of gravity on the body's centre of mass. The exercise conditions were assumed to be identical except for the addition of the Swiss ball. An attempt was made to control for the speed of movement as well. These controls were imparted visually and with simple measurements. It is possible that subtle variations did exist between the conditions but we feel that these differences would not affect the results as they would be overlapped by the natural variability that is seen in EMG recording during any well controlled exercise.

Of practical interest are the low values for muscle activity during the exercises. For the population studied it implies that these exercises are insufficient to produce improvements in strength. Adaptations in terms of endurance or motor control are possible. A less athletic population may achieve strength benefits from these exercises.

## Conclusion

The muscular response during push up exercises on unstable surfaces is task and muscle dependent. When the hands are supported by the Swiss ball (but not the feet) increases in muscle activity can be seen with a greater number of muscles affected.

## Competing interests

The author(s) declare that they have no competing interests.

## Authors' contributions

GL: Conception, design, data collection, data analysis, manuscript preparation

BM, IM, MC, MF: design, data collection, manuscript preparation

**Figure 1:**
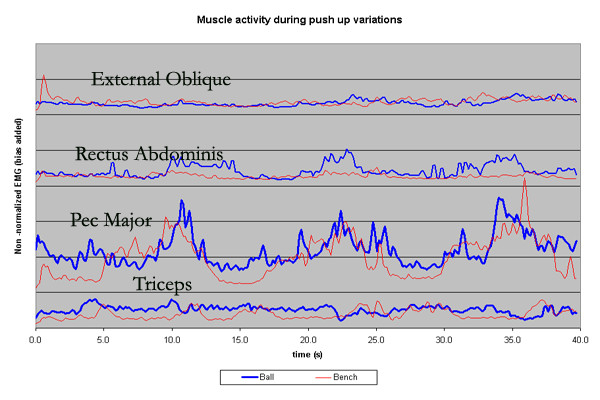
EMG linear envelopes during a push ups with the hands on an exercise bench and a Swiss ball. EMG activity is non-normalized and is therefore in arbitrary EMG units. A bias was added to the activity of the pectoralis major, rectus abdominis and external oblique for ease of viewing to prevent overlap of the muscle's linear envelopes. Three pushups occurred over the course of 30 seconds.

**Figure 2:**
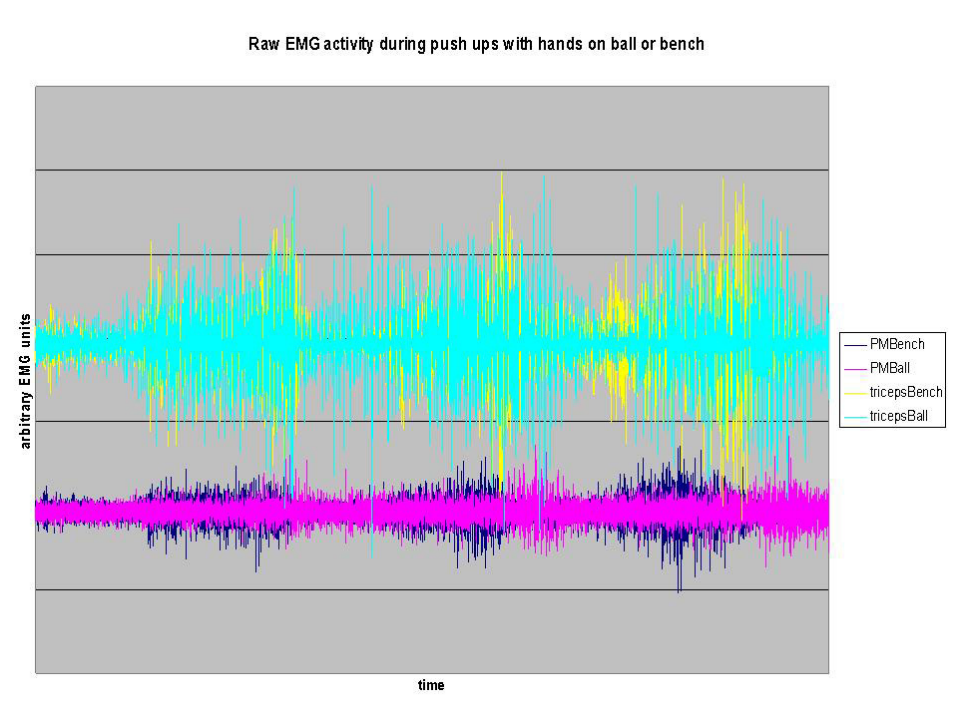
Raw EMG during three push ups with the hands on an exercise bench or a swiss ball for the triceps and pectoralis major muscle. PMBench = Pectoralis Major EMG on bench, PMBall = Pectoralis Major EMG on ball. Bias added to triceps activity for ease of viewing. Trial was 30 seconds in length.

**Figure 3:**
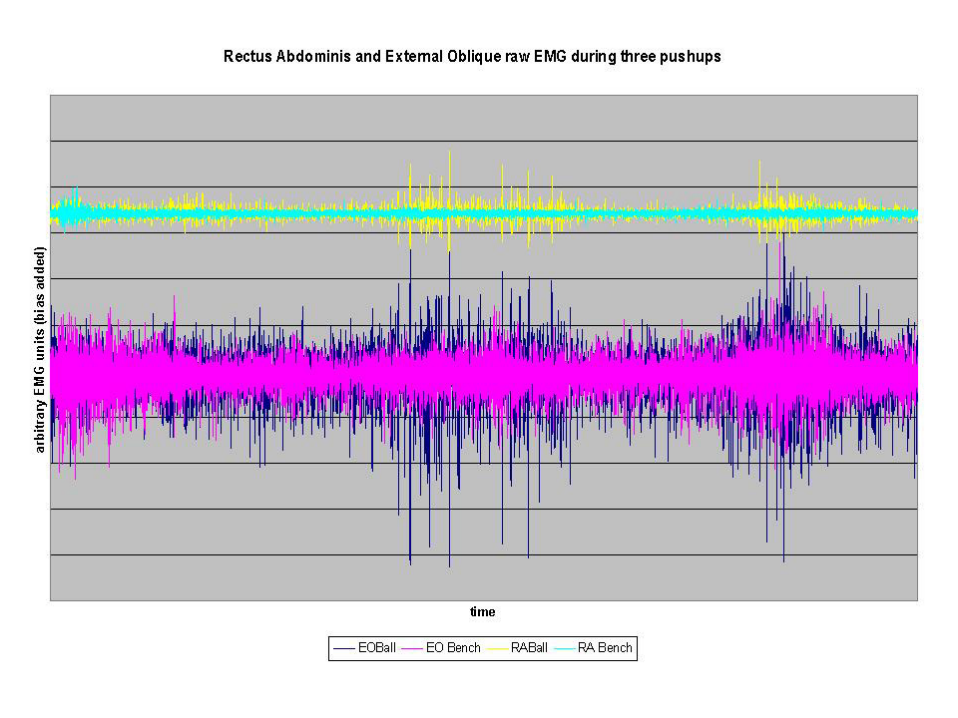
Raw EMG for Rectus Abdominis and External Oblique muscles during three push ups with the hands on or off a Swiss ball. EO = External Oblique, RA = Rectus Abdominis.
